# Editorial: Diet quality, socioeconomic differences, and health disparities

**DOI:** 10.3389/fnut.2023.1250439

**Published:** 2023-07-11

**Authors:** Hoirun Nisa, Kayo Kurotani

**Affiliations:** ^1^Department of Public Health, Faculty of Health Sciences, Syarif Hidayatullah State Islamic University, Jakarta, Indonesia; ^2^Faculty of Food and Health Sciences, Showa Women's University, Tokyo, Japan

**Keywords:** diet quality, dietary behavior, socioeconomic indicators, socioeconomic differences, socioeconomic status, health disparities

Efforts to reduce health disparities have been made since a major recognition of health disparities emerged two decades ago, resulting in overtime improvements in population health. Thus far, however, many health disparities have remained and extended in some cases (1). Evidence suggests that differences in socioeconomic status, whether measured by income, education attainment, occupational position, or area deprivation, are associated with large disparities in health status (2, 3). The association persists across the life course (4, 5) and impacts life expectancy (6). Socioeconomic status appears to significantly affect mortality and morbidity caused by non-communicable diseases (NCDs) such as cardiovascular diseases, cancers, and type 2 diabetes (7). It has also been shown to affect dietary behavior, a major risk factor for NCDs. Worldwide, high intake of sodium, low intake of whole grains, and low intake of fruits are the leading dietary risk factors for mortality and disability-adjusted life-years (8). In addition, the recent COVID-19 pandemic has exacerbated the differences in socioeconomic status and diet quality of the people (9). Although many studies have reported the associations of socioeconomic differences with dietary behavior and health status, the evidence across different populations remains insufficient.

The goal of this Research Topic is to showcase recent studies across different populations relating to impacts of socioeconomic differences in dietary behavior and health disparities.

In this Research Topic, the selected papers focus on the extent of socioeconomic differences in food purchasing practices, expenditure, and consumption (Were et al.), the indicators of socioeconomic status as predictors of animal food consumption (Klink et al.), and the age and cohort trends of the impact of socioeconomic status on dietary diversity in older adults (Yu et al.). In addition, the papers elaborate the role of food assistance programs in supporting diet quality during the COVID-19 pandemic (Lee et al.), the trend of the chronic kidney disease associated with high sodium intake by socio-demographic index (SDI) from 1990 to 2019 (Liu et al.), and the socioeconomic determinants and inequalities in exclusive breastfeeding among children (Hernández-Vásquez and Vargas-Fernández).

It is well supported by epidemiologic data that socioeconomic status has a strong influence on diet quality, with higher socioeconomic status groups have healthier and more sustainable dietary behaviors (10, 11). Indeed, healthy diets with whole grains, lean meats, fish, low fat dairy products, and fresh vegetables and fruit are more likely to be consumed by groups of higher socioeconomic status. Economic considerations substantially affect food choices and are particularly evident among the less wealthy. Low income restricts the ability to buy healthy foods and limits the access to food retailers where healthy food can be purchased more cheaply (10). The study of Were et al. showed that individuals from the poorest households in Kenya were less likely to consume healthy diets with fruits and vegetables and less likely to achieve adequate dietary diversity, while the wealthier households spent significantly more money on food purchases.

A range of socioeconomic indicators have been used to assess the associations of socioeconomic differences with dietary behavior (12). Each indicator reflects a different underlying social process and is likely to make independent and unique contributions to the dietary outcomes (13). Klink et al. evaluated the predictive power of the most used socioeconomic indicators based on two large representative studies in Germany and showed that educational attainment was the strongest and the most consistent socioeconomic indicator for adults' animal food consumption compared to income and occupational status. In older adults, higher socioeconomic status may offset the trend of decreasing dietary diversity due to decreased chewing and digestive functions (14, 15). The study of Yu et al. revealed that Chinese older adults who lived in urban areas and had higher socioeconomic status generally had higher dietary diversity score, although the positive effect of urban residence on dietary diversity score decreased with age and increased across successive cohorts.

In addition, economic and food supply chain shocks during the COVID-19 pandemic have caused substantial increases in the numbers of individuals experiencing food insecurity (16). Adults experiencing food insecurity in Massachusetts, generally with lower income, reported greater disruption in diet during the pandemic with less food consumption (Lee et al.). Evidently, participation in the safety net programs have increased their food consumption, regardless of healthfulness (Lee et al.).

Effective monitoring and management of dietary habits, such as sodium intake, are cost-effective for the prevention and intervention of chronic diseases, including chronic kidney disease (CKD) (17, 18). Regions with different socio-demographic index (SDI) quintiles, a summary measure of socioeconomic development, show discrepancies in the global CKD burden attributable to high sodium intake, reflecting socio-spatial inequalities in CKD prevention, health care, and sodium reduction measures (Liu et al.).

Socioeconomic differences also occur in exclusive breastfeeding practices, a healthy practice that improves both maternal and infant wellbeing; the socioeconomic disparities observed for breastfeeding may be one component contributing to differences in health at various points across the life course (19). In the inequality analysis performed by Hernández-Vásquez and Vargas-Fernández, exclusive breastfeeding in Peruvian children 6 to 59 months of age was concentrated among the poorest mothers and the major contributors were residing in the highlands and jungle. Most Peruvian women living in the highlands and jungle regions have low socioeconomic levels, which could be associated with low exposure to advertising and marketing of breast milk substitutes and where cultural practices increase exclusive breastfeeding (Hernández-Vásquez and Vargas-Fernández).

In summary, socioeconomic status continues to have a major effect on dietary behavior and health status across different populations. Despite all the existing literature and evidence related to this significant topic, the papers in this Research Topic clearly show that many aspects remain to be clarified and understood regarding the associations of socioeconomic differences with dietary behavior and health status across different populations. It is expected that the evidence collected here will be referenced in future food policies to improve diet quality and reduce health disparities.

## Author contributions

HN wrote the article. All authors contributed to the article and approved the submitted version.
